# Obstetric, Neonatal, and Clinical Outcomes of Day 6 vs. Day 5 Vitrified-Warmed Blastocyst Transfers: Retrospective Cohort Study With Propensity Score Matching

**DOI:** 10.3389/fendo.2020.00499

**Published:** 2020-08-04

**Authors:** Dong Soo Park, Ji Won Kim, Eun Mi Chang, Woo Sik Lee, Tae Ki Yoon, Sang Woo Lyu

**Affiliations:** Department of Obstetrics and Gynecology, Fertility Center of CHA Gangnam Medical Center, CHA University School of Medicine, Seoul, South Korea

**Keywords:** blastocyst, embryo transfer, *in vitro* fertilization, day 5 blastocyst transfer, day 6 blastocyst transfer, delayed blastulation, live birth rate, pregnancy outcome

## Abstract

Despite the large number of studies on blastocyst transfers, it is unclear whether day 6 blastocysts have similar pregnancy rates and safety with day 5 blastocysts. Thus, this study aimed to compare the obstetric, neonatal, and clinical outcomes of day 5 and day 6 vitrified blastocyst transfers (VBT). In this retrospective cohort study with propensity score matching, we evaluated 1,313 cycles of VBT performed between January 2014 and December 2015 at the Fertility Center of CHA Gangnam Medical Center. All cycles underwent natural endometrial preparation. We used propensity score matching to compare day 5 and day 6 VBTs in a matched comparison. After propensity score matching, there were 465 cycles of day 5 VBT and 155 cycles of day 6 VBT. Implantation rate (IR), clinical pregnancy rate (CPR), and live birth rate (LBR) were significantly lower in day 6 VBTs (44.2 vs. 53.1%, *p* = 0.023; 48.4 vs. 60.4%, *p* = 0.009; 33.5 vs. 51.8%, *p* < 0.001). Miscarriage rate was significantly higher in day 6 VBTs (29.3 vs. 10.7%, *p* < 0.001). Rate of multiple gestations was similar between the two groups (29.3 vs. 30.2%, *p* = 0.816). Assessing 241 and 52 babies from day 5 and day 6 VBTs, no differences were found in neonatal outcomes including rates of low birth weight, preterm birth, and congenital malformations. In propensity score-matched analysis, obstetric, and neonatal outcomes between day 5 and day 6 VBTs were similar so that day 6 VBTs are as safe as day 5 VBTs. IR, CPR, and LBR were are all significantly lower in day 6 VBTs. Therefore, if there are no differences in the morphological grade between day 5 and day 6 blastocysts, transfer of day 5 vitrified blastocysts should be considered first.

## Introduction

As techniques for *in vitro* fertilization (IVF) and embryo culture have become advanced, many IVF centers can transfer embryos at the blastocyst stage. Some embryos have reached blastocyst stage by day 5 and others not until day 6 or even day 7. Recently, a study reported that the blastulation rate was 66% on day 5, 29% on day 6, and 6% on day 7 (1). Compared with normally growing embryos, there were increased number of abnormal mitotic spindle (2), decreased expression of mitotic spindle (3), and more molecular abnormalities (4) in growth-retarded embryos. These phenomena have raised the question: Does blastocysts with a delayed blastulation maintain acceptable pregnancy rates with safety?

To answer this question, several studies have compared IVF outcomes of day 5 and day 6 blastocyst embryo transfers. However, results of these studies are conflicting. In fresh IVF cycles, many studies suggest that day 5 blastocysts give rise to higher pregnancy rates than day 6 blastocysts (5–8). Theoretically, controlled ovarian hyperstimulation advances endometrial maturation by 1–2.5 days compared with the expected chronological date from oocyte retrieval. It causes asynchronous uterine environment with poor endometrial receptivity and may decrease pregnancy rates (9–11). However, it is difficult to determine whether poor endometrial receptivity is due to impaired embryo quality of day 6 blastocysts or asynchronous uterine environment with poor endometrial receptivity (5, 12).

Therefore, most available studies explore frozen-thawed blastocyst transfer (FBT) cycles or vitrified-warmed blastocyst transfer (VBT) cycles that have a more synchronized endometrium using artificial or medicated endometrial preparations. However, these studies also have conflicting results. Some studies suggest that day 6 blastocysts have comparable clinical outcomes with day 5 blastocysts (5, 13–18), while other studies suggest that clinical outcomes are better with day 5 blastocysts (19–26).

Despite the large number of studies on this field, it is unclear whether day 6 blastocysts have similar pregnancy rates and safety with day 5 blastocysts. Above all, no well-matched cohort study has been conducted yet. Therefore, in this study, we aimed to compare pregnancy and neonatal outcomes of day 5 and day 6 blastocysts in VBT cycles with propensity-score matching.

## Materials and Methods

### Patient Characteristics

We performed a retrospective cohort study to evaluate the outcomes of 1,313 VBT cycles of women under 40 years between January 2014 and December 2015 at the Fertility Center of CHA Gangnam Medical Center. Pregnancy outcomes, including neonatal data, are recorded continuously in the CHA Gangnam Medical Center database. In cases of missing data, telephone surveys were conducted.

**Figure 1:**
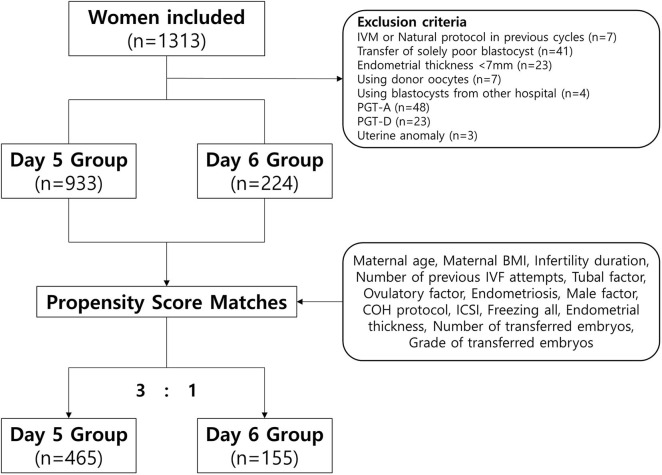
Flowchart of the inclusion and exclusion of participants in this study.

Among 1,313 VBT cycles, we excluded women who underwent VBT using donor oocytes, a single poor-quality blastocyst, blastocysts that underwent a preimplantation genetic test (PGT), blastocysts from other IVF centers, and those who underwent a natural protocol, modified natural protocol, or *in vitro* maturation protocol in previous fresh cycles. We also excluded women who had a thin endometrium (<7 mm) or uterine anomalies. Finally, we included 1,157 VBT cycles (Figure 1).

All VBT cycles were divided into two groups according to the day of blastulation: day 5 group (blastulation on day 5 of culture) and day 6 group (blastulation on day 6 of culture). The study was approved by the Institutional Review Board of CHA Gangnam Medical Center (IRB approval number: GCI 18–30). Informed consent was waived because of the retrospective study design.

### Embryo Culture and Grading

To suppress ovulation until follicle maturity was attained, patients were treated with either a gonadotropin-releasing hormone (GnRH) agonist or a GnRH antagonist. The final follicular maturation was triggered with human chorionic gonadotropin or a GnRH agonist when the mean diameter at least two leading (largest and second largest) follicles was 18 mm. Oocytes were retrieved 36 h later by transvaginal ultrasound-guided needle aspiration of follicles.

Conventional IVF or intracytoplasmic sperm injection (ICSI) was used for embryo fertilization. Fertilization was confirmed when two pronuclear (2PN) zygotes were observed after 16–18 h in ICSI and 18–20 h in conventional IVF. Cleavage-stage embryos were cultured in a cleavage medium (Cook, Queensland, Australia), while blastocyst-stage embryos were cultured on a blastocyst medium (Cook). Embryos were cultured in HERA cell 240 incubators (Thermo Fisher Scientific, MA, USA) in an environment with 5% O_2_, 6% CO_2_, at 37°C. The oil-drop culture method was applied using a four-well dish (NUNC™, Thermo Fisher Scientific). At 10-μl drops, the embryos were cultured individually to observe their development. Then, light paraffin oil (OVOIL, Vitrolife AB, Sweden) was dropped onto the media to prevent the medium from drying and undergoing fast pH change.

Blastocysts were morphologically graded according to the Gardner classification, which takes into its expansion, inner cell mass (ICM) constitution, and trophectoderm composition prior to freezing on day 5 or 6 (27). Blastocysts had good quality if all of the following criteria were met: the blastocyst expanded and filled the embryo completely (grade 3), the ICM was composed of several loosely grouped cells (grade B), and the trophectoderm contained few cells that formed loose epithelium (grade B). Blastocysts had poor quality if any of these criteria were not met.

### Vitrification and Warming of Blastocysts

For blastocysts vitrification, artificial shrinkage was performed on all blastocysts, and assisted hatching was facilitated with a laser. The blastocysts were pre-equilibrated in hydroxyethyl piperazine-ethanesulfonic acid (HEPES) medium (Quinn's-HEPES; SAGE, *in-vitro* Fertilization, Inc.) supplemented with 7.5% ethylene glycol and 7.5% dimethyl sulfoxide (Sigma-Aldrich, St. Louis, MO, USA) for 2.5 min and then transferred in 15% ethylene glycol, 15% dimethyl sulfoxide, and 0.5-M sucrose for the final equilibration. Thereafter, the blastocysts were loaded onto an electron microscopic (EM) gold grid (EM Grid, SPI Supplies) using a fine glass pipette. The EM grids containing the blastocysts were immediately plunged into slush liquid nitrogen using VitMaster, a vitrification device (IMT Ltd., Ness Ziona, Israel).

For vitrified blastocysts warming, the EM grids were sequentially transferred to culture dishes containing HEPES medium supplemented with 0.5-, 0.25-, 0.125-, and 0.0625-M sucrose at 2.5-min intervals, with 20% human serum albumin (SAGE BioPharma, Bedminster, NJ). The vitrified-warmed blastocysts were washed with blastocyst medium (Cook Medical, Bloomington, IN, USA) in a 37°C environment with 6% CO_2_, 5% O_2_, and 89% N_2_ and then cultured overnight.

### VBT Protocol

All women underwent natural endometrial preparation. They were closely monitored for signs of dominant follicle collapse by transvaginal ultrasonography from days 10 to 12 of the menstrual cycle. Ovulation was confirmed if the follicular wall lost its clear appearance (28). Then, luteal support was initiated using Crinone 8% w/w Progesterone Vaginal Gel (Merck Serono Ltd., Middlesex, UK) or vaginal progesterone Utrogestan 600 mg (Hanhwa Pharmaceuticals, Seoul, Korea). An embryo replacement catheter (Cook) was used, and the warmed blastocysts were transferred under abdominal ultrasound guidance on day 5 after ovulation was observed. Finally, the embryo transfer catheter was checked to confirm that the embryo was no longer in the catheter.

### Outcome Measures

Clinical and obstetric outcomes were as follows: implantation rate (IR), clinical pregnancy rate (CPR), multiple pregnancy rate (MPR), ectopic pregnancy rate, miscarriage rate, live birth rate (LBR). IR was calculated as the number of gestational sacs seen by ultrasonography divided by the total number of transferred blastocysts. Clinical pregnancy was defined as the presence of a fetal heartbeat on ultrasonogram. Miscarriage was defined as the spontaneous cessation of a clinical pregnancy before 20 gestational weeks. LBR was defined as delivery of a viable infant at >28 gestational weeks. Neonatal outcomes were as follows: birth weight, gestational age at delivery, and presence of malformations.

### Statistical Analyses

We compared pregnancy and neonatal outcomes for the day 5 and day 6 groups in a propensity score-matched cohort to minimize potential biases (Figure 1) (29). The propensity scores were calculated using binary logistic regression analyses based on the following patient and menstrual cycle variables at baseline: maternal age, maternal body mass index, infertility duration, number of previous IVF attempts, presence of tubal factor (as diagnosed by tubal obstruction, tubal adhesion, or previous salpingitis), polycystic ovarian syndrome, endometriosis, male factors (defined as oligoasthenoteratozoospermia or sperm concentration <15 × 10^6^/mL, vitality <40%, motility <32%, normal morphology <4%), protocol of controlled ovarian hyperstimulation, intracytoplasmic sperm injection, use of a freeze-all strategy, endometrial thickness, number of transferred embryos, and quality of transferred embryos. The matched ratio for day 5 vs. day 6 was 3:1. Quantitative variables are expressed as means ± standard deviations (SD) and were analyzed using Student's *t*-test. Qualitative variables are expressed as frequencies and percentages and were analyzed using the χ^2^-test. All statistical analyses were performed using SPSS version 23 (IBM). *P* < 0.05 was considered to indicate statistical significance.

## Results

### Patient Demographics and Previous IVF Cycle Characteristics

**Figure 2:**
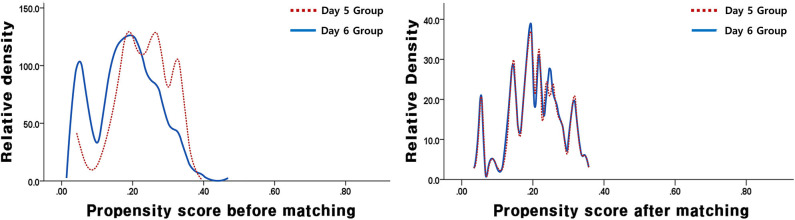
The distribution of propensity scores before and after matching.

In this study, a total of 1,157 VBT cycles were analyzed, including 933 VBT cycles of day 5 group and 224 VBT cycles of day 6 group. Day 5 and 6 VBT cycles were matched at 3:1, that is, 155 triplet cycles in day 5 vs. day 6 cohorts (Figure 1). The distribution of propensity scores before and after matching is shown in Figure 2. The demographic characteristics of the patients are presented in Table 1. No statistical difference was found in maternal age at the time of oocyte retrieval between the two groups. All other variables such as previous IVF attempts and etiology of infertility were comparable. Previous IVF cycle characteristics are summarized in Table 2. The number of 2PN zygote decreased in the day 6 group (*p* = 0.048), and the number of freezing blastocysts was lower in the day 6 group (*p* = 0.007).

**Table 1:** Characteristics of vitrified-warmed blastocyst transfer cycles by day of blastulation.

	**Day 5 (*N* = 465)**	**Day 6 (*N* = 155)**	***p*-value**
Maternal age (years)	34.4 ± 2.6	34.7 ± 2.8	0.260
Paternal age (years)	35.7 ± 4.2	36.0 ± 4.6	0.438
Maternal BMI	21.2 ± 3.0	21.3 ± 2.8	0.886
Infertility duration (years)	3.7 ± 2.2	3.5 ± 2.2	0.450
Previous IVF attempts (*n*)	1.7 ± 1.2	1.8 ± 1.2	0.725
AMH (ng/mL)	3.80 ± 3.88	4.07 ± 4.30	0.473
Basal FSH (mIU/mL)	5.01 ± 4.05	5.01 ± 4.03	0.995
**Etiology of infertility (%)**			
Tubal	134/465 (28.8)	47/155 (30.3)	0.760
Ovulatory	87/465 (18.7)	29/155 (18.7)	1.000
Endometriosis	21/465 (4.5)	6/155 (3.9)	0.824
Male	168/465 (36.1)	50/155 (32.3)	0.437

*Data are presented as mean ± SD and proportion (%)*.

*AMH, anti-Müllerian hormone; BMI, body mass index; FSH, follicle-stimulating hormone; LH, luteinizing hormone*.

**Table 2:** Characteristics of previous fresh *in vitro* fertilization cycles by day of blastulation.

	**Day 5 (*N* = 465)**	**Day 6 (*N* = 155)**	***p-*value**
Cycle day (day)	12.9 ± 5.3	12.5 ± 1.1	0.339
E2 on hCG (pg/mL)	3,000.2 ± 1,368.1	2,909.3 ± 1,379.3	0.294
Ovarian stimulation protocol (%)			0.830
Antagonist	443/465 (95.3)	147/155 (94.8)	
Agonist	22/465 (4.7)	8/155 (5.2)	
Retrieved oocytes (*n*)	16.6 ± 8.1	15.6 ± 7.5	0.184
Intracytoplasmic sperm injection (%)	233/465 (50.1)	71/155 (45.8)	0.404
2PN (*n*)	12.3 ± 5.8	11.2 ± 5.2	0.048
Freezing blastocyst (*n*)	4.5 ± 3.3	3.7 ± 2.9	0.007
Freezing all (%)	41/465 (8.8)	13/155 (8.4)	0.869

*Data are presented as mean ± SD and proportion (%)*.

*E2, estradiol; hCG, human chorionic gonadotropin; 2PN zygote, two pronuclear zygote*.

### Clinical, Obstetric, and Neonatal Outcomes of VBT Cycles

The clinical and obstetric outcomes of the VBT cycles are shown in Table 3. The IR, CPR, and LBR were significantly lower in the day 6 group than in the day 5 group (IR 44.2 vs. 53.1%, *p* = 0.023; CPR 48.4 vs. 60.4%, *p* = 0.009; LBR 33.5 vs. 51.8%, *p* < 0.001). The miscarriage rate was higher in the day 6 group than in the day 5 group (29.3 vs. 10.7%, *p* < 0.001). No significant differences were found in the ectopic pregnancy rate (1.3 vs. 2.8%, *p* = 0.458) and MPR (29.3 vs. 30.2%, *p* = 0.816). Table 4 displays the neonatal outcomes. No significant differences were observed in gestational age (days) at birth (268.8 d ± 16.6 d vs. 266.4 d ± 14.5 d, *p* = 0.306) and birth weight (3,030.6 g ± 562.3 g vs. 2,897.1 g ± 597.4 g, *p* = 0.099) between the two groups. Rates of preterm birth (PTB), low birth weight (LBW), and congenital malformations were all comparable (PTB 15.4 vs. 21.2%, *p* = 0.402; LBW 16.2 vs. 22.1%, *p* = 0.333; congenital malformations 0 vs. 0.6%, *p* = 0.549, respectively).

**Table 3:** Clinical and obstetric outcomes of vitrified-warmed blastocyst transfer cycles by day of blastulation.

	**Day 5 (*N* = 465)**	**Day 6 (*N* = 155)**	***p-*value**
Endometrial thickness (mm)	9.6 ± 1.9	9.6 ± 1.6	0.972
Transferred blastocyst (*n*)	1.7 ± 0.8	1.7 ± 0.7	0.701
Double embryo transfer (%)	358/465 (77.0)	125/155 (80.6)	0.342
Grade			0.546
Two good blastocysts	156/465 (33.5%)	58/155 (37.4%)	
One good + One poor blastocysts	202/465 (43.4%)	67/155 (43.2%)	
One good blastocyst	107/465 (23.0%)	30/155 (19.4%)	
Implantation (%)	361/685 (53.1)	95/215 (44.2)	0.023
Odds ratio (95% CI)	Reference	0.700 (0.514–0.952)	
Clinical pregnancy (%)	281/465 (60.4)	75/155 (48.4%)	0.009
Odds ratio (95% CI)	Reference	0.614 (0.426–0.885)	
Live birth (%)	241/465 (51.8)	52/155 (33.5%)	<0.001
Odds ratio (95% CI)	Reference	0.469 (0.321–0.686)	
Ectopic pregnancy (%)	8/281 (2.8)	1/75 (1.3%)	0.458
Odds ratio (95% CI)	Reference	0.461 (0.057–3.746)	
Miscarriages (%)	30/281 (10.7)	22/75 (29.3%)	<0.001
Odds ratio (95% CI)	Reference	3.473 (1.859–6.487)	
Multiple gestation	85/281 (30.2)	22/75 (29.3%)	0.816
Odds ratio (95% CI)	Reference	0.936 (0.534–0.639)	

*There were no double embryo transfers with mixed day 5 and day 6 blastocysts. Data are presented as mean ± SD and proportion (%)*.

**Table 4:** Neonatal outcomes of vitrified-warmed blastocyst transfer cycles by day of blastulation.

	**Day 5 (*N* = 241)**	**Day 6 (*N* = 52)**	***p-*value**
Gestational days at birth	266.4 ± 14.5	268.8 ± 16.6	0.306
PTB per cycles (%)	51/241 (21.2)	8/52 (15.4)	0.402
Odds ratio (95% CI)	Reference	0.706 (0.312–1.598)	
Gestational weeks at birth			0.089
Extremely preterm (<28 weeks)	0/241 (0.0)	1/52 (1.9)	
Very preterm (28–32 weeks)	4/241 (1.7)	0/52 (0)	
Moderate to late preterm (32–37 weeks)	47/241 (19.5)	7/52 (13.5)	
Normal (More than 37 weeks)	190/241 (78.8)	44/52 (84.6)	
Birth weight (g)	2,897.1 ± 597.4	3,030.6 ± 562.3	0.099
LBW per live births (%)	72/326 (22.1)	12/74 (16.2)	0.333
Odds ratio (95% CI)	Reference	0.716 (0.363–1.412)	
Proportion of birth weight			0.368
Extremely LBW (<1,000 g)	0/326 (0)	0/74 (0)	
Very LBW (1,000–1,500 g)	7/326 (2.1)	0/74 (0)	
LBW (1,500–2,500 g)	65/326 (19.9)	12/74 (16.2)	
Normal (More than 2,500 g)	254/326 (77.9)	62/74 (83.8)	
Congenital malformations	2/326 (0.6)	0/74 (0)	0.549

*Data are presented as mean ± SD and proportion (%)*.

*PTB, preterm birth; LBW, low birth weight*.

## Discussion

In this study, we compared the pregnancy and neonatal outcomes of day 5 and day 6 blastocysts in VBT cycles. Our present result indicates that VBT cycles with day 6 blastocysts were significantly inferior to those with day 5 blastocysts in terms of IR, CPR, and LBR. Additionally, the miscarriage rate was higher in VBT cycles with day 6 blastocysts. However, no significant differences were found in the neonatal outcomes between the two groups. Considering these findings, if there were no differences in the morphological grade between day 5 and day 6 blastocysts, transfer of day 5 vitrified blastocysts should be considered first.

Our results are consistent with previous studies that reported significantly lower clinical outcomes from FBT or VBT cycles with day 6 blastocysts (19–24). Among them, Ferreux et al. (23) reported a significantly lower LBR of day 6 blastocysts, regardless of the grades of embryos. Baseline characteristics were not significantly different between the study groups, and the blastocysts have similar grades using the grading scale proposed by Gardner et al. (27). However, they did not match the two groups, although the day 5 group had three times more cycles than the day 6 group. Moreover, our miscarriage rates in the day 5 and day 6 groups were similar to those in their study. Haas et al. also reported a significantly lower clinical outcomes including IR and CPR (22). Interestingly, they compared day 5 blastocysts with good-quality day 6 blastocysts (≥3 BB). However, they warmed day 5 blastocysts 20–24 h before the embryo transfer, while they warmed day 6 blastocysts 2–4 h prior to embryo transfer.

Considering the lower clinical outcomes of day 6 blastocysts, there is still a degree of controversy in previous studies (5, 13–18). Yang et al. (15) reported that high-quality (≥3 BB) day 6 blastocysts in VBT had similar developmental potential and pregnancy outcomes to those of high-quality day 5 blastocysts. However, they did not match day 5 and 6 groups, although the day 5 group had five times more cycles than the day 6 group. Moreover, they did not report LBR. In a meta-analysis, Sunkara et al. (18) compared the clinical outcomes of FBT with day 5 blastocysts and those with day 6 blastocysts. They included 2,502 cycles from 15 controlled studies and concluded that FBT with day 6 blastocysts have similar CPR and LBR to FBT with day 5 blastocyst, if the morphological grade is the same. However, this meta-analysis had clinical heterogeneity and limited consideration of the confounders in the included studies.

Although we did not perform preimplantation genetic testing for aneuploidy, chromosomal abnormality could explain the significant difference in clinical outcomes including miscarriage rate between day 5 and 6 VBT groups. Indeed, there have been studies reporting that slower developing blastocysts have higher aneuploidy rate (25, 26). Taylor et al. (26) reported that day 5 blastocysts had a higher chance of being euploid than day 6 blastocysts. The risk of aneuploidy of day 6 blastocysts was 10% higher than that of day 5 blastocysts. To reduce bias, they used a sibling embryo model, that is, they included patient who had biopsy on both day 5 and day 6 blastocysts in the same IVF cycles. From time-lapse culture systems, some studies revealed the close relationship between timely cell division and developmental competence with kinetic data in accordance with our results (30–32). Campbell et al. (33) reported that embryos having single or multiple aneuploidy had delayed initiation of blastulation compared with euploid embryos in time-lapse culture systems.

An increase of spindle abnormalities in day 6 blastocysts could explain our significant results. Hashimoto et al. (2) conducted a cytoskeletal analysis of day 5 and 6 blastocysts. They found that the incidence of abnormal spindles was significantly higher in day 6 blastocysts, and IR and CPR were significantly higher in VBT of day 5 blastocysts. Interestingly, they evaluated the incidence of chromosomal abnormalities of the abortus and reported no differences between day 5 and 6 groups. They hypothesized that most blastomeres with abnormal spindles are eliminated before implantation. This hypothesis may support the safety of VBT with day 6 blastocysts, as revealed in our results.

In our study, the MPR of day 5 VBT was similar with that of day 6 VBT. In the day 5 VBT group, 2 good blastocyst transfers occurred in 42.8% (57/133) of multiple pregnancies, and one good and one poor blastocyst transfer occurred in 32.5% (28/86) of multiple pregnancies. Similarly, day 6 VBT had a 37.8% (14/37) of multiple pregnancies with 2 good blastocysts transfers and 29.1% (7/24) of multiple pregnancies with one good and one poor blastocyst transfer. This suggests that the quality of embryos, as well as the day of blastulation, is important for clinical outcomes. This is consistent with past reports that the morphological grades of embryos are one of the most important prognostic factors in IVF (34–37).

In contrast to the centers at which previous studies were conducted, our center used a natural endometrial preparation. Compared with artificial or medicated endometrial preparations, the clinical outcomes of VBT with natural endometrial preparations are not to be inferior (38, 39). Artificial endometrial preparations have been linked to a high miscarriage rate (40, 41). In addition, IVF is covered by the national health insurance system in Korea, so it is possible to perform daily ultrasonograms at low cost. For natural endometrial preparations, some clinicians use serial LH tests. However, the role of serial LH monitoring with ultrasonogram has been a subject of much debate in natural endometrial preparations and there is no clear definition of or consensus regarding LH surge (42–44). Furthermore, serial LH tests are not covered by the national health insurance. Because of these reasons, our IVF center prefers to perform natural endometrial preparations without serial LH tests.

The primary strength of our study is that we performed analysis using propensity score matching to control for potential confounders between the study groups. It is impossible to perform randomized controlled study for comparing day 5 and 6 blastocysts; thus, a prospective observational study or well-designed matched study is more appropriate. Therefore, a propensity score matching analysis, like this study, would be an adequate design for comparing day 5 blastocysts with day 6 blastocysts. Propensity score matching analysis is used for observational studies wherein allocation is not random, and it can also be viewed as an approach seeking to replicate the random assignment of study populations in conventional randomized controlled trials (45). Additionally, we included VBT cycles that are able to promote better embryo-endometrial synchrony, so we eliminate endometrial receptivity bias from controlled ovarian hyperstimulation. Moreover, this study has a single-center design; therefore, all IVF cycles were done under uniform conditions, and embryos were cultured in the same media using the same techniques by the same embryologists. By this, we can minimize not only observational biases but also the influence of varying culture media on neonatal birth weights (46, 47). Finally, all our patients were of the same ethnicity.

This study has some limitations. In performing propensity score matching, which controls for multiple confounding variables, the sample size decreased from 1,313 cycles to 620 cycles. This increases the risk of a type 2 error. Since the study had a retrospective design, there were not enough data that could be associated with PTB and LBW, including a previous history of PTB or LBW, underlying maternal disease, or pregnancy-associated diseases. In addition, we did not include data from VBTs with single poor-quality or double poor-quality blastocysts. Although there were 41 cases of VBT with a single poor-quality blastocyst during the study period, this number was considered too small to be included.

In conclusion, with the ever-evolving IVF technology, especially vitrification-warming technique, defining safety, and clinical outcomes of growth-retarded blastocysts compared with timely growing blastocysts is essential. Although VBT with day 6 blastocysts can lead to acceptable clinical outcomes and safety, our propensity score-matched study suggests that day 5 blastocysts for VBT offer significant favorable clinical outcomes by reducing miscarriage rate, if the morphological grades are not different between day 5 and 6 blastocysts. In the future, well-designed prospective study, especially focusing on the euploidy of growth-retarded blastocysts, is needed.

## Data Availability Statement

The raw data supporting the conclusions of this article will be made available by the authors, without undue reservation.

## Ethics Statement

The study was approved by the Institutional Review Board of CHA Gangnam Medical Center (IRB Approval Number: GCI 18–30). Informed consent was waived because of the retrospective study design.

## Author Contributions

SL contributed to the conception and design. DP, JK, and EC collected data and conducted analysis. WL and TY were responsible for data interpretation. DP drafted the manuscript. All authors read and approved the final manuscript.

## Conflict of Interest

The authors declare that the research was conducted in the absence of any commercial or financial relationships that could be construed as a potential conflict of interest.
